# Nifedipine Potentiates Susceptibility of *Salmonella* Typhimurium to Different Classes of Antibiotics

**DOI:** 10.3390/antibiotics10101200

**Published:** 2021-10-01

**Authors:** David Haschka, Manuel Grander, Johannes Eibensteiner, Stefanie Dichtl, Sabine Koppelstätter, Günter Weiss

**Affiliations:** Department of Internal Medicine II, Medical University of Innsbruck, 6020 Innsbruck, Austria; david.haschka@i-med.ac.at (D.H.); manuel.grander@student.i-med.ac.at (M.G.); johannes.eibensteiner@mail.de (J.E.); sdichtl@biochem.mpg.de (S.D.); sabine.koppelstaetter@tirol-kliniken.at (S.K.)

**Keywords:** iron, nifedipine, azithromycin, lipocalin 2, *Salmonella*

## Abstract

The calcium channel blocker nifedipine induces cellular iron export, thereby limiting the availability of the essential nutrient iron for intracellular pathogens, resulting in bacteriostatic activity. To study if nifedipine may exert a synergistic anti-microbial activity when combined with antibiotics, we used the mouse macrophage cell line RAW267.4, infected with the intracellular bacterium *Salmonella* Typhimurium, and exposed the cells to varying concentrations of nifedipine and/or ampicillin, azithromycin and ceftriaxone. We observed a significant additive effect of nifedipine in combination with various antibiotics, which was not observed when using *Salmonella,* with defects in iron uptake. Of interest, increasing intracellular iron levels increased the bacterial resistance to treatment with antibiotics or nifedipine or their combination. We further showed that nifedipine increases the expression of the siderophore-binding peptide lipocalin-2 and promotes iron storage within ferritin, where the metal is less accessible for bacteria. Our data provide evidence for an additive effect of nifedipine with conventional antibiotics against *Salmonella,* which is partly linked to reduced bacterial access to iron.

## 1. Introduction

Due to increasing numbers of infection with multi-resistant Gram-negative bacteria, new antibiotics or effective combinations of existing anti-microbial drugs are urgently needed to tackle this enormous health concern [1,2]. In this regard, iron emerges as a potential therapeutic target, as it is an essential growth factor for both the host and the pathogen [3,4]. As a sufficient availability of iron for microbes is needed for their proliferation and pathogenicity, host response strategies attempt to limit iron accessibility for invading microbes, for which the term nutritional immunity has been coined [5,6,7]. Of interest, specific and partly different pathways for microbial iron restriction are induced by the immune system, depending on pathogen localization, with respect to the cellular habitat (intra- versus extracellular) or with regard to tissue-specific aspects [3,8,9].

In addition, iron has subtle effects on cell-mediated immune effector pathways. For example, iron inhibits the activity of interferon-γ (IFN-γ)-driven effector pathways of macrophages, such as tumor necrosis factor α and nitric oxide formation, resulting in a reduced immune response to intracellular pathogens [10,11,12]. Nevertheless, it must be emphasized that a certain amount of iron is important for the generation of oxygen radicals by macrophages via the Fenton reaction [13], but also for differentiation, proliferation and mobilization of lymphocytes or neutrophils [14,15].

Recently, we showed that the calcium channel blocker nifedipine mobilizes tissue iron in mouse models of iron overload; this was linked to stimulation of iron transport via divalent metal transporter 1 (Dmt1) [16]. Subsequently, we demonstrated that nifedipine increases host resistance to infection with the intracellular bacterium *Salmonella* enterica serovar Typhimurium (S.tm.) by limiting iron access to microbes within macrophages, resulting in a bacteriostatic effect [17]. Nifedipine is used mainly as an antihypertensive and antianginal drug. It inhibits the entry of calcium ions by blocking voltage-gated L-type calcium channels in vascular smooth muscle and cardiac myocytes. Reduction of intracellular calcium decreases peripheral arterial vascular resistance and results in dilation of coronary arteries. This, in turn, leads to a reduction in systemic blood pressure and increased oxygen supply to the myocardium. The plasma concentrations achieved have been described as 115 +/− 7 ng/mL [18]. The most common adverse effects include flushing, peripheral edema, dizziness and headache. Tolerability is better with extended-release preparations than with immediate-release preparations of nifedipine [19].

In case of infection with intracellular bacteria such as S.tm., macrophage re-program their iron metabolism by increasing the expression of the only known iron exporter ferroportin (Fpn1) to promote iron egress, but also express several host resistance proteins aiming to limit iron access for bacteria, such as lipocalin-2 (Lcn2), which binds bacterial enterobactin-type siderophores, or the iron binding protein lactoferrin or natural resistance-associated macrophage protein 1 (Nramp1), which induces transcellular iron shifting [20,21,22,23,24,25]. Accordingly, the protective mechanism of nifedipine exerted in mice was paralleled by increased expression of Fpn1 in the spleen, whereas splenic levels of the iron storage protein ferritin and serum iron concentrations were reduced [9]. 

S.tm. is a facultative intracellular Gram-negative bacterium capable of persisting and replicating within host macrophages. The latest edition of the White-Kauffmann-Le Minor classification describes 2500 serovars of *Salmonella* belonging to the five different species [26]. *Salmonella* is a very common foodborne pathogen and of great public health importance, causing a total of 93.8 million cases of non-typhoid gastroenteritis worldwide [27]. The intracellular proliferation of *Salmonella* is highly dependent on iron, and therefore *Salmonella* has developed different mechanisms, including the expression of siderophores like enterobactin and salmochelins or of high-affinity transmembrane iron transporters, to secure a sufficient supply with iron. Based on our observations of an effect of nifedipine on iron homeostasis and *Salmonella* proliferation, we questioned whether nifedipine, a well-known antihypertensive drug used for decades in clinical medicine, would exert an additive effect to conventional antibiotic treatment, thereby increasing the efficacy of anti-microbial therapy.

We therefore established an infection model with S.tm. in the murine macrophage cell line RAW267.4, investigating the effect of nifedipine and various antibiotics on the course of infection and toward modulation of iron homeostasis. 

## 2. Results

Our aim was to investigate different antibiotics with regard to an additive anti-microbial effect in combination with nifedipine. For this purpose, it was necessary to find appropriately comparable antibiotic concentrations in a cell culture model with murine macrophage RAW267.4 cells. We therefore infected RAW264.7 cells with *Salmonella* Typhimurium (S.tm.) and treated them with ampicillin, azithromycin or ceftriaxone in different concentrations (Supplementary Materials Figure S1). From these curves, we calculated the concentration for a 50% bacterial elimination-dose (ED50). This should ensure that the antibiotic concentrations used lead to a significant reduction of bacteria but still leave enough room to study for a possible additive effect of nifedipine. Subsequently, the doses were set based on the calculated ED50 for ampicillin at 0.07 µg/mL, azithromycin 0.3 µg/mL and ceftriaxone 0.005 µg/mL, and these were used as standard doses for all further experiments. Nifedipine was used at a dose of 17.3 μg/mL (50 µmol/L), which has been shown to be effective in previous investigations [17].

### 2.1. Additive Effect of Different Antibiotics and the Calcium Channel Inhibitor Nifedipine

**Figure 1:**
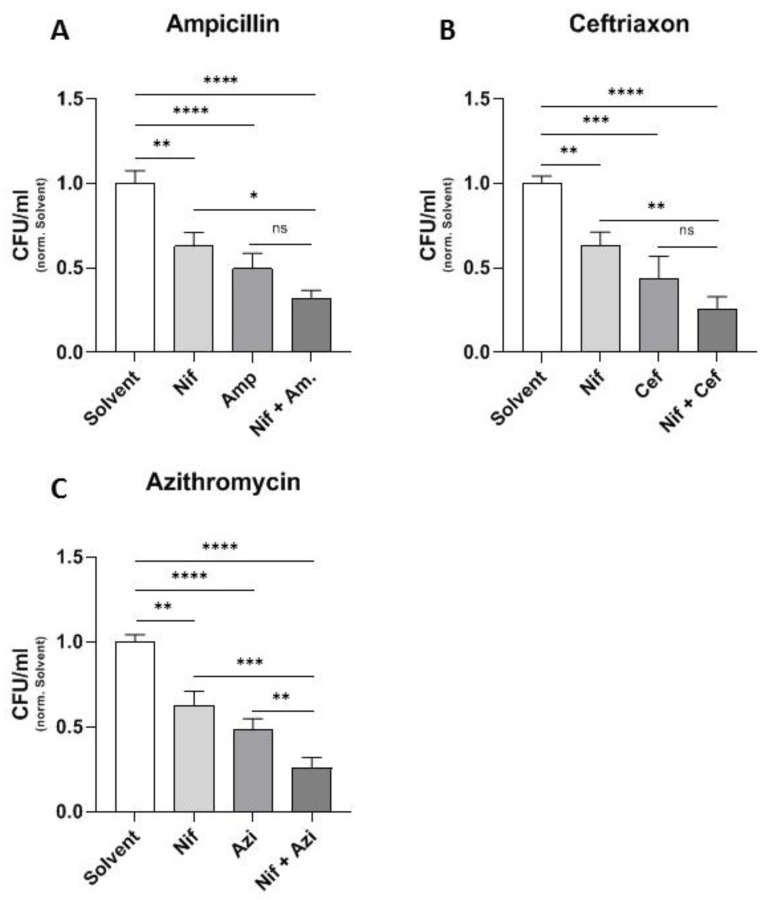
Different antibiotics in combination with nifedipine. RAW264.7 cells were infected with *Salmonella* Typhimurium for 24 h and treated with 17.3 μg/mL nifedipine (Nif) as well as one of the indicated antibiotics: ampicillin (Amp, 0.07 μg/mL) (**A**), ceftriaxon (Cef, 0.005 μg/mL) (**B**) or azithromycin (Azi, 0.3 μg/mL) (**C**). Data are expressed as mean ± SEM of at least four independent experiments. Superscripts indicate statistical significance as follows: * *p* < 0.05, ** *p* < 0.01, *** *p* < 0.001, **** *p* < 0.0001.

In a next step, we attempted to identify possible combinations of antibiotics and nifedipine. In all experiments, nifedipine, when used alone, was able to significantly reduce bacterial numbers in comparison to a solvent control (Figure 1A–C). As expected, antibiotics significantly reduced bacterial loads within macrophages in that experimental setting. Of interest, the combination of all antibiotics with nifedipine led to a synergistic effect. However, the combination with the bacteriostatic antibiotic azithromycin led to the highest relative reduction of CFUs in comparison to the solvent control (Figure 1C). We therefore decided to use azithromycin for a more detailed analysis of the underlying mechanism because of its great effectiveness and consistency.

### 2.2. Dose-Response Curve of Azithromycin and Nifedipine

**Figure 2:**
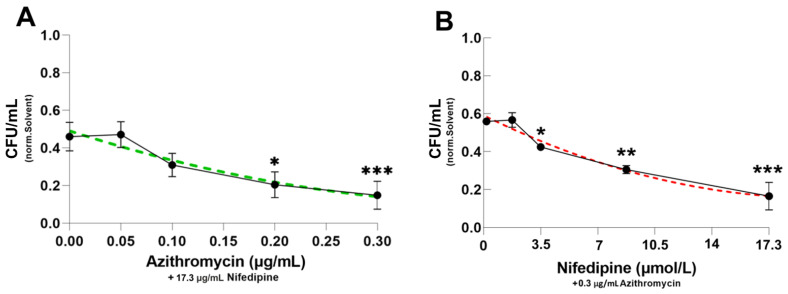
Dose-response curves of nifedipine and azithromycin. RAW264.7 cells were infected with *Salmonella* Typhimurium for 24 h and treated with nifedipine and azithromycin in indicated concentrations. In (**A**) nifedipine concentration was constant at 17.3 μg/mL, while azithromycin concentrations were between 0 and 0.3 μg/mL. (**B**) Azithromycin concentration was constant at 0.3 μg/mL, while nifedipine concentrations were between 0 and 17.3 μg/mL. Data are expressed as mean ± SEM of at least four independent experiments. Superscripts indicate statistical significance as follows: * *p* < 0.05, ** *p* < 0.01, *** *p* < 0.001.

We then preformed dose-response curves to further verify the effects of the combination of azithromycin and nifedipine. This is particularly important because concentrations of nifedipine should be as low as possible due to its antihypertensive effects. The increasing concentrations of azithromycin in the presence of a constant nifedipine dose (17.3 μg/mL) resulted in a progressive reduction of bacterial counts with approximately linear kinetics (Figure 2A). In a comparable fashion, nifedipine dose dependently increased the antimicrobial activity of a fixed dose of azithromycin (Figure 2B). Interestingly, 10 µmol/L of nifedipine was able to further reduce bacterial load significantly when compared to treatment with azithromycin alone. Overall, nifedipine supplementation exerts an even stronger additional effect than escalation of azithromycin dosages. 

### 2.3. Nifedipine Has No Direct Antibacterial Activity

**Figure 3:**
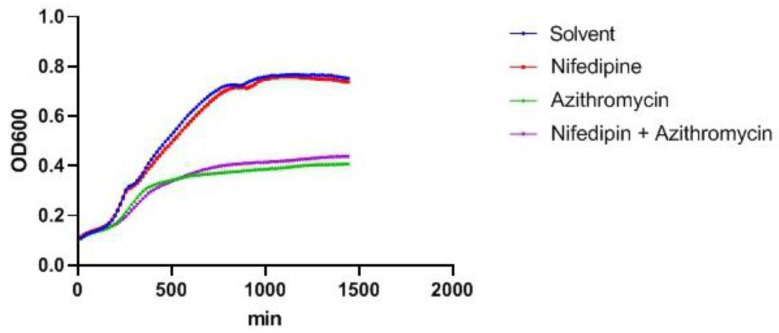
Bacterial proliferation assay. Bacterial cultures with *Salmonella* Typhimurium in DMEM medium were incubated with solvent control, 17.3 μg/mL nifedipine, 0.3 μg/mL azithromycin or a combination for the indicated time period. Bacterial proliferation was measured photometrically by OD600 every 15 min. One representative experiment of three is shown.

Subsequently, we addressed possible mechanisms for these additive anti-microbial effects. For this purpose, studies for a possible direct anti-microbial effect of nifedipine on bacteria were performed. A proliferation assay with bacteria in DMEM medium plus azithromycin and/or nifedipine over time showed that nifedipine exerts no direct anti-bacterial activity, as it did not show any difference in proliferation kinetics as compared to the solvent control (Figure 3). In contrast, azithromycin strongly reduced bacterial proliferation kinetics, which were not altered by the addition of nifedipine. 

### 2.4. Effect of Hepcidin on Antibacterial Activity of Nifedipine against Intramacrophage Salmonella and Mutant Salmonella with Iron Acquisition Defects

**Figure 4:**
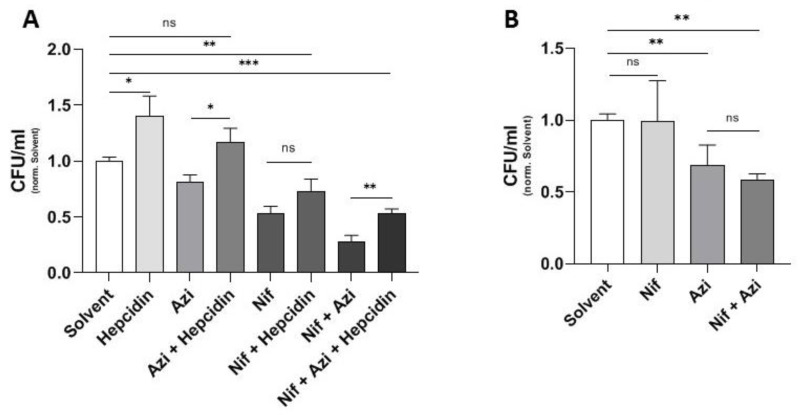
Effect of hepcidin on antibacterial activity of nifedipine against intramacrophage *Salmonella* and mutant *Salmonella* with iron acquisition defects. In (**A**) RAW264.7 cells were infected with *Salmonella* Typhimurium for 24 h and treated with nifedipine (17.3 μg/mL), azithromycin (0.3 μg/mL) and/or hepcidin (1 μg/mL), as indicated. In (**B**) RAW264.7 cells were infected with *Salmonella* Typhimurium lacking the three main iron acquisition systems (∆entC, ∆sitABCD, ∆feo) and treated with nifedipine (17.3 μg/mL) and/or azithromycin (0.3 μg/mL). Data are expressed as mean ± SEM of at least four independent experiments. Superscripts indicate statistical significance as follows: * *p* < 0.05, ** *p* < 0.01, *** *p* < 0.001.

As shown earlier by the authors, nifedipine leads to upregulation of Fpn1 in the spleen in vivo, resulting in a reduction of spleen iron content, which was suggested to be responsible for improved control of *Salmonella* infection growth [17]. We thus studied the effects of azithromycin and nifedipine in S.tm. infected RAW264.7 macrophages in the presence and absence of hepcidin. The peptide hepcidin is the master regulator of iron homeostasis and exerts its action on iron metabolism by binding to Fpn1, resulting in internalization and degradation of the latter [28]. Accordingly, exposure of cells to hepcidin blocks iron export and increases intracellular iron levels and intra-macrophage bacterial numbers [29], as is also shown here (Figure 4A). Such an effect was also seen when infected cells were treated with azithromycin or nifedipine or a combination of both, indicating that an increased bacterial access to iron increases the resistance of *Salmonella* to nifedipine or azithromycin (Figure 4A). 

To underscore the importance of iron for nifedipine-mediated antibacterial activity, we performed experiments using RAW264.7 cells infected with *Salmonella* Typhimurium lacking three important iron acquisition systems (∆entC, ∆sitABCD, ∆feo). Herein no significant effect of nifedipine treatment, either when used alone or in combination with azithromycin, could be observed (Figure 4B), indicating the crucial importance of nifedipine-mediated modulation of bacterial iron access for its bacteriostatic activity. Nevertheless, azithromycin was able to reduce bacterial numbers in a comparable manner as in infection with wild-type *Salmonella* (Figure 4B). 

### 2.5. Modulation of Iron Homeostasis by Nifedipine in Infected Macrophages

**Figure 5:**
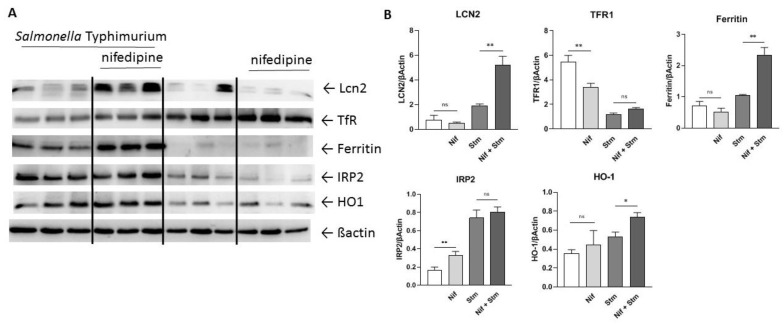
Regulation of iron-related proteins. RAW264.7 cells were infected with *Salmonella* Typhimurium for 24 h and treated with nifedipine (17.3 μg/mL) as indicated. (**A**) Western Blot of Tfr1, ferritin, Lcn2, HO1, Irp and βactin. (**B**) Quantification of the Western Blot. Data are expressed as mean ± SEM. One representative Western Blot of three independent experiments is shown. Superscripts indicate statistical significance as follows: * *p* < 0.05, ** *p* < 0.01.

In order to see how nifedipine impacts macrophage iron homeostasis and intracellular iron availability for bacteria, we studied the expression of critical iron genes in RAW264.7 macrophages by Western Blot. While heme oxygenase 1 (Ho1) was low in uninfected macrophages, it was increased upon infection, indicating intracellular oxidative stress. This was also paralleled by an increased expression of the bacterial siderophore-binding peptide lipocalin-2 (Lcn2) (Figure 5). Importantly, Lcn2 expression was significantly higher in nifedipine treated *Salmonella*-infected macrophages as in those treated with solvent. When studying indicators of intracellular iron availability, we found that transferrin receptor (TfR) expression was high in uninfected macrophages and then decreased in infected cells, which would be indicative either for higher intracellular iron levels or an inhibitory effect of the pathogen or inflammatory cytokines on TfR expression [30,31]. Therefore, we determined iron regulatory protein 2 (Irp2) levels, as its expression is post-translationally regulated by the availability of metabolically active intracellular iron [32]. Of note, Irp2 levels were increased in *Salmonella*-infected cells, indicating reduced levels of metabolically accessible iron, but Irp levels were not different with/without nifedipine treatment in infected cells. This was paralleled by a marked increase of the iron storage protein ferritin in infected macrophages, where iron is efficiently stored and not accessible for bacteria like *Salmonella*. Our observation indicated that the effect of nifedipine is partly linked to limiting iron availability for intramacrophage *Salmonella*, which can be referred to increased expression of the siderophore-binding protein Lcn2 and efficient iron storage within ferritin. 

## 3. Discussion

A wide variety of therapeutic approaches are being investigated in the search for new antimicrobial therapies, including manipulation of host or microbial iron homeostasis, as iron is an essential nutrient for bacteria [33,34,35,36]. This study provides evidence for a synergistic effect of antibiotics and the calcium channel blocker nifedipine on *Salmonella* Typhimurium survival in RAW267.4 cells. Of interest, nifedipine has been shown to alter iron homeostasis and exert bacteriostatic effects [17]. The additive effects observed herein were most pronounced with the combination of nifedipine and azithromycin as compared to azithromycin alone. Notably, ampicillin and ceftriaxone were not as efficient in combination with nifedipine to reduce bacterial numbers. In contrast to macrolides such as azithromycin, beta-lactam antibiotics do not accumulate within cells where intracellular bacteria such as *Salmonella* have their habitat. These effects should have been aligned in the first dose-finding experiments. 

The putative mechanism of antibiotic action of nifedipine, particularly the targeted retention of iron for intracellular bacteria, argues for a bacteriostatic mode of action. A possible explanation for the stronger additive effect could be the combination of two bacteriostatic drugs. This has also already been described for antibiotic combinations [37]. However, enhanced activity of antibiotics by the addition of calcium channel inhibitors is also thought, in part, to be due to inhibition of P-glycoprotein and efflux pumps in bacterial species, allowing antibiotics to accumulate intra-bacterially [38,39]. This mode of action has been only shown for verapamil, a calcium channel blocker with a different chemical structure. Additionally, calcium channel blocker might directly interfere with bacterial sodium and/or other calcium channel blockers as shown before [40,41], influencing their physiological processes [42]. 

While putative effects of calcium antagonists on bacteria by modulating electrolyte homeostasis have been described for different species and in various models [43], we could not find a direct effect of nifedipine on proliferation kinetics of S.tm. Rather, we showed that nifedipine exerts anti-bacterial activity largely by modulating host iron homeostasis and bacterial access to iron. Strikingly, we were able to show for the first time a significant induction of lipocalin-2 (Lcn2) expression by nifedipine. The induction of the bacterial siderophore-sequestering peptide Lcn2 with immunomodulatory effects might explain in part the bacteriostatic effects of nifedipine. The protective effect of Lcn2 against *Salmonella* Typhimurium infection through immunomodulation and iron restriction has been previously demonstrated [44,45,46]. Thus, the induction of Lcn2 would argue for a bacteriostatic effect of nifedipine, as observed herein [47]. This antimicrobial effect of Lcn2 is not restricted to S.tm. and has also been shown for other Gram-negative bacteria, including *Klebsiella*, E.coli or *Chlamydia* [44,48,49].

As shown earlier, nifedipine may restrict iron availability for intracellular bacteria via the induction of ferroportin expression, with a subsequent increase of iron export. This mechanism has been proposed as one of the key mechanisms to starve intracellular bacteria of iron [4,21]. In a line with this, we could show that the addition of hepcidin, which reduced Fpn1 expression and macrophage iron export, significantly increased bacterial numbers within macrophages, and treatment with azithromycin, nifedipine or their combination became less effective, pointing to the essential role of a sufficient supply of iron for bacterial resistance and pathogenicity [50]. Moreover, nifedipine treatment of infected macrophages increased ferritin levels but increased intracellular Irp2, indicating a reduction of metabolically active iron in the cytoplasm and therefore limited iron availability for intracellular bacteria. 

## 4. Materials and Methods

### 4.1. Cell Culture

RAW264.7 (murine macrophage) cells were obtained from the American Type Culture Collection and maintained in Dulbecco’s modified eagles medium (DMEM; purchased from Lonza, Basel, Switzerland) containing 10% fetal calf serum (FCS; PAN Biotech, Aidenbach, Germany), 2 mM l-glutamine (Lonza) and 1% penicillin–streptomycin (Lonza) at 37 °C in humidified air containing 5% CO_2_. Cells were seeded in dishes and grown overnight until 70–80% confluent.

### 4.2. Bacterial Strain and Salmonella Infection of Macrophages

Wild-type (wt) *Salmonella* Typhimurium strain ATCC 14028 as well as a mutant form of the same strain lacking all three main iron acquisition systems (∆entC, ∆sitABCD, ∆feo) were used for all experiments and grown in LB broth medium (Sigma, St. Louis, MO, USA) to late-logarithmic phase. Before in vitro infection, cells were washed three times with phosphate-buffered saline (PBS; Lonza) and incubated in complete DMEM without antibiotics.

RAW264.7 macrophages were infected at a multiplicity of infection (MOI) of 10 for 1 h at 37 °C as previously described [21]. After 1 h, cells were washed three times with PBS, and complete DMEM containing 25 mg/mL of gentamicin (Life Technologies, Carlsbad, CA, USA) was added in order to kill extracellular bacteria. For quantification of intracellular *Salmonella* by means of gentamicin protection assay, macrophages were lysed and plated in appropriate dilutions onto LB agar plates.

For experiments involving nifedipine (Sigma), cells were exposed to this substance at a final concentration of 17.3 μg/mL (50 μmol/L) one hour after infection with bacteria was performed. Antibiotics were used in the following concentrations: azithromycin (Sigma) 0.3 μg/mL, ampicillin (Sigma) 0.07 μg/mL and ceftriaxone (Sigma) 0.005 μg/mL, or as indicated in the text. Hepcidin (Peptanova, Sandhausen, Germany) was used in a concentration of 1 μg/mL. For solvent control, DMSO was used. Addition of the mentioned components took place after 1 h of infection, and if combined, they were administered simultaneously. 

Proliferation assay bacteria were cultured overnight in LB medium. At an OD600 of 0.5, bacteria were counted and 2 million *Salmonella* were cultured and stimulated with the indicated components in DMEM (Lonza) with 1% FCS (Biochrom) in 96-well plates. OD600 was then measured every 15 min. 

### 4.3. Western Blot

Protein extraction and Western Blotting were performed as described [51]. Used antibodies were a rabbit anti-Lcn2 antibody (1:1000; abcam, Cambridge, United Kingdom), a mouse anti-TFR1 antibody (1:1000; Sigma Aldrich), a rabbit anti-ferritin antibody (1:500; Sigma), a rabbit anti-Ho1 antibody (1:1000; abcam), a rabbit anti-Irp2 antibody (1:1000; Novusbio, Littleton, CO, USA) and a rabbit actin antibody (1:500; Sigma Aldrich), and appropriate HRP-conjugated secondary antibodies (1:2000, anti-rabbit; 1:4000, anti-mouse; Dako, Glostrup, Denmark). For quantification, densitometry data were acquired on a ChemiDoc Touch Imaging System (Bio-Rad, Hercules, CA, USA) and analyzed with Image Lab 5.2.1. (Bio-Rad).

### 4.4. Statistical Analysis

Statistical analysis was performed using a GraphPad Prism software package. Results were expressed as mean ± SEM. Statistical tests included unpaired two-tailed Student’s *t*-test and one-way ANOVA followed by Bonferroni-Holmes Multiple Comparison Test. *p* values of 0.05 or less were considered to denote significance.

## 5. Conclusions

Taken together, our results demonstrate that nifedipine exerts bacteriostatic activity against infection with the intracellular bacterium S.tm. and that this calcium antagonist increases the anti-microbial potential of conventional antibiotics when used in combination. We also demonstrated that an increase of intracellular iron levels enhanced bacterial resistance to innate immune responses, antibiotics and nifedipine, pointing to the importance of host and bacterial iron homeostasis for the course of infections.However, nifedipine is an antihypertensive drug, and thus modified pharmacological agents, which on the one hand can affect bacterial iron availability and exert bacteriostatic activity while on the other hand have no hypotensive potential, could be a valuable addition to the arsenal of effective anti-microbial drugs to target the challenge of increasing anti-microbial resistance. 

## Data Availability

All data generated or analyzed during this study are included in this published article and its Supplementary Materials.

## Supplementary Materials

The following are available online at https://www.mdpi.com/article/10.3390/antibiotics10101200/s1, Figure S1: Dose-response effects of ampicillin, ceftriaxone and azithromycin on *Salmonella* survival.
