# Supplementary material for: Nifedipine Potentiates Susceptibility of Salmonella Typhimurium to Different Classes of Antibiotics
Source: Antibiotics (Basel). 2021 Oct 1;10(10):1200. doi: 10.3390/antibiotics10101200 (PMC8532624; doi:10.3390/antibiotics10101200)
Supplement: Supplementary file 1 [file antibiotics-10-01200-s001.zip › antibiotics-1402770-supplementary.pdf]

Supplemental Materials

Nifedipine Potentiates Susceptibility of *Salmonella* Typhimurium to Different Classes of Antibiotics

David Haschka †, Manuel Grander †, Johannes Eibensteiner, Stefanie Dichtl, Sabine Koppelstätter and Günter Weiss *

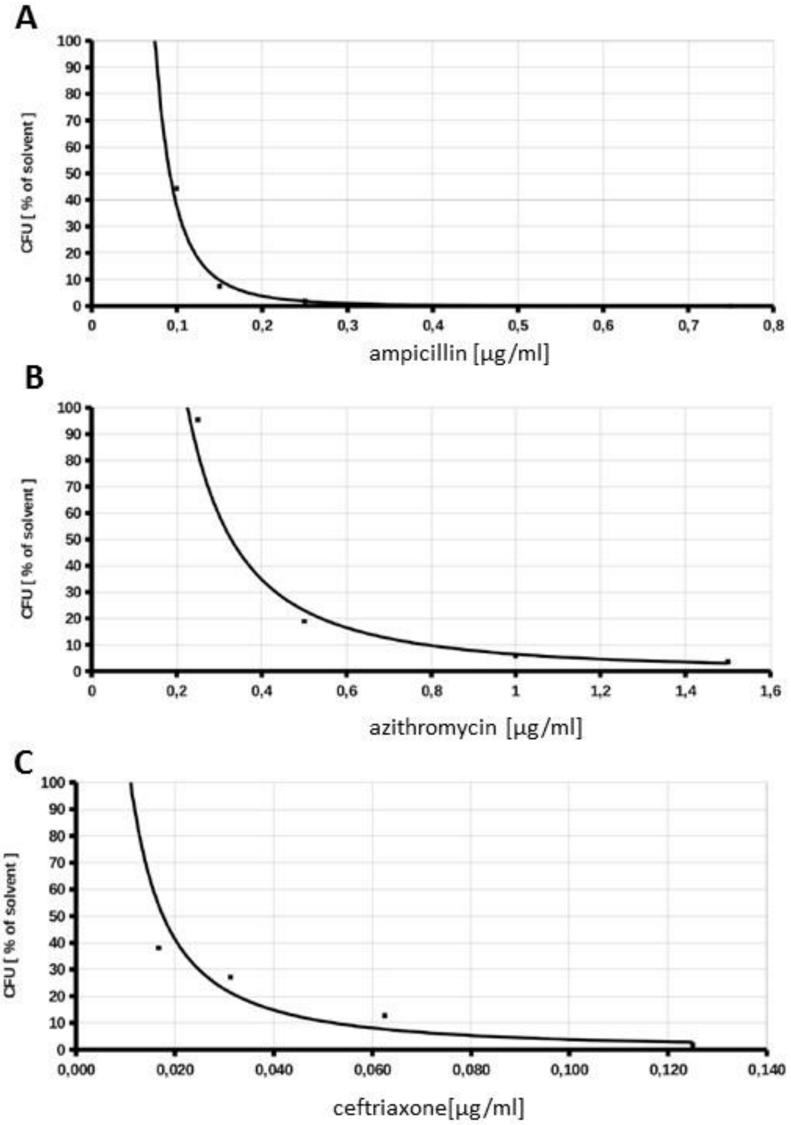

Figure S1. Dose-response effects of ampicillin, ceftriaxone and azithromycin on *Salmonella* survival. RAW264.7 cells were infected with *Salmonella* Typhimurium for 24 hours and treated ampicillin (**A**), azithromycin (**B**) and ceftriaxone (**C**) in the indicated concentrations. CFU were determined by plating on LB agar.
